# High-Flow Nasal Cannula Outcomes in COVID-19-Associated Acute Hypoxemic Respiratory Failure: Illness Severity, Awake Prone Positioning, and Risk of Intubation in a Single-Center ICU Cohort

**DOI:** 10.3390/medicina62101984

**Published:** 2026-10-14

**Authors:** Merve Özlem Kılıçaslan, Mert Canbaz, Özlem Polat, Perihan Ergin Özcan, Figen Esen

**Affiliations:** 1 Department of Anesthesiology and Reanimation, Istanbul Faculty of Medicine, Istanbul University, 34093 Istanbul, Türkiye; 2 Department of Anesthesiology and Reanimation, Dr. Ersin Arslan Research and Training Hospital, 27090 Gaziantep, Türkiye; 3 Department of Anesthesiology and Reanimation, Haseki Research and Training Hospital, 34096 Istanbul, Türkiye

**Keywords:** high-flow nasal cannula, awake prone positioning, acute hypoxemic respiratory failure, intensive care unit, intubation, illness severity, COVID-19

## Abstract

*Background and Objectives:* High-flow nasal cannula (HFNC) is widely used in acute hypoxemic respiratory failure, yet predictors of successful noninvasive support and the role of awake prone positioning remain incompletely defined, and observational analyses are vulnerable to immortal-time bias. We evaluated factors associated with HFNC success and the association of awake prone positioning with HFNC outcomes in critically ill adults with COVID-19-associated acute hypoxemic respiratory failure, using analyses designed to reduce time-related and indication-related bias. *Materials and Methods:* In this retrospective, single-center intensive care unit (ICU) cohort study, adults with virologically confirmed COVID-19 who received HFNC, with awake prone positioning considered as part of the initial HFNC strategy, between April 2020 and December 2021 were followed from HFNC initiation until endotracheal intubation or ICU discharge. HFNC success was defined as avoidance of intubation during the index ICU stay. Multivariable logistic regression with multiple imputation of missing covariates, propensity score-based inverse probability of treatment weighting (IPTW), and landmark time-to-event analyses were used to assess the association between awake prone positioning and HFNC failure. *Results:* Among 222 patients, HFNC was successful in 72 (32.4%) and unsuccessful in 150 (67.6%). Failure was associated with lower PaO_2_/FiO_2_ ratios, higher FiO_2_ requirements, and higher APACHE II and SOFA scores at ICU admission (all *p* ≤ 0.011). In the imputed multivariable model, awake prone positioning was associated with lower HFNC failure (odds ratio [OR] 0.23; 95% CI 0.10–0.53; *p* < 0.001), whereas higher APACHE II score (OR 1.09 per point; 95% CI 1.02–1.17; *p* = 0.012), higher HFNC FiO_2_ (OR 1.41 per 10%; 95% CI 1.15–1.73; *p* < 0.001), and lower PaO_2_/FiO_2_ ratio (*p* = 0.037) were independently associated with failure. IPTW yielded a consistent estimate (OR 0.22; 95% CI 0.10–0.47). In time-to-event analyses, the unadjusted association was strong but likely influenced by early deterioration; after a day-1 landmark it attenuated yet remained significant (hazard ratio [HR] 0.52; 95% CI 0.34–0.79; *p* = 0.002), and at a day-2 landmark it was non-significant unadjusted (HR 0.66; 95% CI 0.39–1.11) but significant after adjustment for illness severity (HR 0.47; 95% CI 0.25–0.88; *p* = 0.019). *Conclusions:* Patients undergoing awake prone positioning during HFNC had a lower observed incidence of intubation, although the difference was more modest than crude analyses suggested and sensitive to landmark timing, whereas greater illness severity and more severe hypoxemia at ICU admission were independently associated with failure. These findings support severity-informed monitoring and timely escalation during HFNC. Causality cannot be inferred because of the retrospective design and residual confounding, particularly confounding related to the ability to tolerate prone positioning.

## 1. Introduction

High-flow nasal cannula (HFNC) has become a cornerstone of respiratory support for patients with acute hypoxemic respiratory failure, delivering high and stable fractions of inspired oxygen (FiO_2_) while washing out nasopharyngeal dead space, generating low-level positive airway pressure, and preserving spontaneous breathing and patient comfort [1]. In the landmark FLORALI trial, HFNC reduced 90-day mortality and, among more severely hypoxemic patients, the rate of intubation compared with that of conventional oxygen or noninvasive ventilation [2]. During the COVID-19 pandemic, the surge in hypoxemic respiratory failure accelerated HFNC adoption in intensive care units (ICUs) and generated extensive real-world experience with this modality [3,4]. Although HFNC can avert or delay intubation in selected patients, failure remains common, and delayed escalation to invasive ventilation has been associated with worse outcomes; identifying patients likely to benefit from continued noninvasive support versus those requiring earlier intubation remains a major clinical challenge [4,5]. The respiratory rate–oxygenation (ROX) index, which combines the SpO_2_/FiO_2_ ratio with respiratory rate measured during the first hours of HFNC, is the most widely validated bedside tool for this purpose [5].

Awake prone positioning emerged as a complementary strategy during HFNC. By improving dorsal lung recruitment and ventilation–perfusion matching without exposing patients to ventilator-associated lung injury, awake proning improves oxygenation in spontaneously breathing patients with hypoxemic respiratory failure [6,7]. The randomized meta-trial by Ehrmann and colleagues showed that awake prone positioning reduced treatment failure and intubation in COVID-19 patients supported with HFNC [8], and a subsequent systematic review and meta-analysis reported a reduction in intubation, most consistently in patients receiving HFNC [9]. However, individual randomized trials have been less consistent: the COVI-PRONE and PROFLO trials found no reduction in intubation with awake prone positioning [10,11], and adherence, tolerance, timing, and patient selection have been repeatedly identified as key modifiers [12,13]. The optimal integration of awake prone positioning with HFNC in critically ill patients therefore remains incompletely defined [14].

Crucially, the response to HFNC reflects not only respiratory mechanics but also global illness severity and extrapulmonary organ dysfunction. Baseline physiologic derangement, captured by severity-of-illness scores such as the Acute Physiology and Chronic Health Evaluation II (APACHE II) and the Sequential Organ Failure Assessment (SOFA), is associated with outcomes in severe respiratory failure, including COVID-19 [15,16]. Evidence syntheses of noninvasive oxygenation strategies further emphasize that treatment success depends on timely recognition of patients at risk of failure, not on oxygenation support alone [4,17]. Reliance on oxygenation metrics in isolation may therefore underestimate the risk of HFNC failure in patients with evolving systemic disease.

Against this background, and given that observational studies of an exposure applied *during* follow-up are vulnerable to immortal-time bias [18,19], we conducted a single-center ICU cohort study of HFNC outcomes in COVID-19-associated acute hypoxemic respiratory failure. We focused on the association between awake prone positioning and HFNC success, interpreted within a broader framework of global illness severity, and applied multiple imputation, propensity-score weighting, and landmark time-to-event analyses to reduce indication-related and time-related bias. Our aim was to inform bedside decision-making regarding HFNC initiation, monitoring, and timely escalation in critically ill patients.

## 2. Materials and Methods

### 2.1. Study Design and Setting

This retrospective observational cohort study was conducted in the adult ICU of Istanbul University—Istanbul Faculty of Medicine. The study was approved by the Istanbul University Istanbul Faculty of Medicine Clinical Research Ethics Committee (approval no. 2022/741216; 18 February 2022), and the requirement for individual informed consent was waived because only de-identified clinical data were analyzed. All procedures complied with the Declaration of Helsinki, and reporting followed the Strengthening the Reporting of Observational Studies in Epidemiology (STROBE) statement [20].

### 2.2. Study Population and Eligibility Criteria

Consecutive adult ICU admissions between 1 April 2020 and 31 December 2021 were screened. Inclusion criteria were age ≥ 18 years, laboratory-confirmed COVID-19 by reverse-transcription polymerase chain reaction, and management with HFNC for acute hypoxemic respiratory failure during the ICU stay. Patients admitted outside the study period, those without virologic confirmation, and those who did not receive HFNC were excluded. For patients with more than one ICU admission during the study period, only the first admission was considered.

### 2.3. Data Sources and Variable Definitions

Data were abstracted from the hospital electronic medical record and ICU flow sheets using a standardized collection form. Collected variables included demographics, comorbidities, and chronic home medications. Illness severity at ICU admission was quantified with the APACHE II score (baseline mortality risk) and the SOFA score (organ dysfunction at ICU entry); the maximum SOFA score during the ICU stay and the SOFA score on ICU day 6 were recorded as follow-up variables. Laboratory data comprised routine hematologic, biochemical, inflammatory, coagulation, and cardiac panels obtained at ICU admission. Respiratory support variables included the timing of HFNC initiation and discontinuation, total HFNC duration, and device settings (FiO_2_, flow rate) at ICU admission. Arterial blood gas values (PaO_2_/FiO_2_ ratio), respiratory rate, and laboratory samples were obtained at ICU admission before HFNC initiation, and HFNC FiO_2_ and flow rate represent the initial device settings; APACHE II and SOFA scores were calculated from the worst values within the first 24 h of ICU admission. Because HFNC was started at ICU admission in 215 of 222 patients, these variables reflect the patient’s condition immediately before or at HFNC initiation in the large majority of the cohort. COVID-19-specific treatments administered before ICU admission (hydroxychloroquine, azithromycin, favipiravir, remdesivir, corticosteroids, tocilizumab, and anakinra) were recorded. Calendar time was represented by consecutive ICU admission order. Treatment eras were defined by change-point analysis of treatment practice: corticosteroid use before ICU admission increased abruptly after the 50th admission (from 6.0% to 79.7%), coinciding with the cessation of hydroxychloroquine and azithromycin use and with the adoption of routine awake proning. Patients were therefore classified into an early era (first 50 admissions) and a later era (subsequent 172 admissions). Escalation was captured from ICU documentation, including transition to noninvasive ventilation (when applicable) and endotracheal intubation. Additional organ-support therapies (vasopressor or inotropic support, renal replacement therapy [RRT], extracorporeal membrane oxygenation) were recorded with initiation and discontinuation times. All data were independently extracted by two investigators and cross-checked; discrepancies were resolved by a consensus review of the source records.

### 2.4. Exposure: HFNC Therapy and Awake Prone Positioning

HFNC was the preferred first-line noninvasive support for COVID-19-associated acute hypoxemic respiratory failure in our ICU during the study period; CPAP or noninvasive ventilation was used selectively. HFNC was initiated in patients with persistent hypoxemia despite conventional oxygen therapy at the treating clinician’s discretion, with flow and FiO_2_ titrated to respiratory distress and target oxygenation. Escalation to intubation was based on clinical deterioration despite HFNC optimization (worsening hypoxemia, increasing work of breathing, respiratory fatigue, altered mental status, hemodynamic instability, or inability to protect the airway). No predefined, protocol-based intubation criteria were used during the study period; the decision to intubate was based on bedside clinical judgment by the treating clinician. An SpO_2_ below 85% served as a general alert but did not mandate intubation when the overall clinical picture was stable (e.g., patients with “happy hypoxia” who were comfortable and not using accessory muscles); conversely, patients were promptly intubated if they showed severe work of breathing, accessory muscle use, or persistent distress despite escalation of FiO_2_ or sedation, even when SpO_2_ was relatively preserved. From mid-to-late 2020 onward, awake prone positioning was part of institutional practice and was routinely considered at HFNC initiation (at ICU admission in 215 of 222 patients) and initiated as part of the initial HFNC strategy when clinically feasible; it was not introduced de novo later during the HFNC course, although the exact clock time of the first prone session was not recorded for individual patients. During the first months of the pandemic, before this practice became routine, awake prone positioning was applied more selectively. Awake prone positioning was defined as placement in the prone position while receiving HFNC without invasive ventilation. Contraindications to awake prone positioning were impaired consciousness, hemodynamic instability, abdominal obesity (large waist circumference), and agitation persisting despite dexmedetomidine sedation. No upper limit was set for the duration of individual sessions; the longest tolerated duration was targeted, and sessions were continued during both day and night. Patients able to eat orally were returned to the supine position during meals and for 1 h afterwards and were then re-proned. Repositioning was performed by nurses and patient-care assistants under physician supervision, with pillows used for positioning support. During positioning, SpO_2_, respiratory rate, heart rate, and invasive arterial blood pressure were monitored continuously, and arterial blood gases were obtained routinely four times daily and additionally at the clinician’s discretion. A session was terminated, and the patient returned to the supine position, in the case of a decrease in SpO_2_ of more than 10 percentage points not recovering within a few minutes, increasing respiratory distress without desaturation, hemodynamic deterioration, or severe positional discomfort. Awake prone positioning was discontinued upon clinical improvement, clinical deterioration despite proning, absence of any change in clinical parameters or respiratory effort between the prone and supine positions, or inability to continue because of agitation. Prone positioning applied during invasive mechanical ventilation after intubation was not considered part of the exposure. Patients were classified as exposed if prone positioning was established during this initial HFNC period and as unexposed otherwise; reasons for non-application in individual patients were not systematically documented. Because the ability to tolerate prone positioning is likely related to illness severity, confounding by indication was anticipated and addressed analytically (Section 2.6). Exposure was recorded as a binary variable (yes/no); cumulative exposure was recorded as the total number of documented prone days. Because prone sessions were discontinued once respiratory distress resolved and oxygen requirements decreased, cumulative prone duration reflects the clinical trajectory (improvement or early intubation) rather than the treatment dose, and cumulative exposure was interpreted descriptively only.

### 2.5. Outcomes

The primary outcome was HFNC success, defined as avoidance of endotracheal intubation after HFNC initiation during the index ICU stay. Patients intubated after HFNC initiation during the same admission constituted the HFNC failure group. Secondary outcomes included ICU and hospital length of stay, ICU mortality, and requirement for organ-support therapies. Four patients died in the ICU without intubation; in the time-to-event analyses, these deaths were treated as competing events.

### 2.6. Statistical Analysis

Analyses were performed in R (version 4.5.1; R Foundation for Statistical Computing, Vienna, Austria), IBM SPSS Statistics (version 27; IBM Corp., Armonk, NY, USA), and Python (version 3.12; Python Software Foundation, Wilmington, DE, USA; statsmodels 0.15 and lifelines 0.30). Continuous variables are presented as mean ± standard deviation or median (interquartile range [IQR]) and categorical variables as counts (percentages). Groups were compared using the Student *t*-test or Mann–Whitney *U* test for continuous variables and the χ^2^ or Fisher exact test for categorical variables, as appropriate. Baseline characteristics were also compared according to awake prone positioning, and standardized mean differences (SMDs) were reported. All tests were two-sided, with *p* < 0.05 considered significant. Comparisons across the extensive admission biomarker panel were exploratory and not adjusted for multiplicity; associated *p*-values should be interpreted as hypothesis-generating.

**Missing data.** Body mass index (BMI), respiratory rate, and SOFA score were missing in 33–36% of patients; other model covariates were missing in ≤3.2%. Missingness for each variable, overall and according to outcome and exposure, is reported in Supplementary Table S1. A recorded HFNC FiO_2_ of 0 in one patient was considered implausible and set as missing. To avoid the loss of power and potential selection bias of complete-case analysis, missing values were handled by multiple imputation by chained equations (m = 25 imputations), with estimates pooled by Rubin’s rules [21]. The imputation model included the outcome (HFNC failure); all covariates of the multivariable and propensity-score models (age, sex, BMI, APACHE II score, SOFA score, PaO_2_/FiO_2_ ratio, HFNC FiO_2_, respiratory rate, chronic heart failure, chronic kidney disease, and awake prone positioning); the additional covariates of the sensitivity analyses (mean arterial pressure, heart rate, Charlson Comorbidity Index (CCI), and corticosteroid, tocilizumab, and anakinra treatment before ICU admission); and admission order. All variables with missing data were continuous and were imputed by predictive mean matching with five donors. Each imputation was generated in an independent chain of 20 iterations with a fixed random seed. Convergence was assessed by visual inspection of trace plots of the mean and standard deviation of imputed values across iterations, which showed adequate mixing without systematic trends (Supplementary Figure S1). Complete-case analysis was performed as a sensitivity check, and its results are reported in Supplementary Table S2.

**Multivariable models.** Multivariable logistic regression identified factors independently associated with HFNC failure. Covariates were pre-specified on clinical grounds rather than univariable significance, and comprised age, sex, BMI, APACHE II score, PaO_2_/FiO_2_ ratio, HFNC FiO_2_, respiratory rate, chronic heart failure, chronic kidney disease, and awake prone positioning. HFNC FiO_2_ was modeled per 10% increment to yield a stable, interpretable estimate. A sensitivity model replaced APACHE II with SOFA. To ensure that covariates reflected the patient’s condition at HFNC initiation, all multivariable, propensity-score, and landmark analyses were repeated after excluding the 7 patients in whom HFNC was not started at ICU admission. To address potential confounding by clinical stability, an extended model additionally adjusted for mean arterial pressure, heart rate, Charlson Comorbidity Index, and corticosteroid and tocilizumab treatment before ICU admission. To address temporal changes in COVID-19 management, further sensitivity analyses (i) additionally adjusted the multivariable, IPTW, and landmark analyses for calendar time, modeled as admission order using a restricted cubic spline with four knots; (ii) additionally adjusted the multivariable model for calendar time and corticosteroid, tocilizumab, and anakinra treatment before ICU admission; and (iii) restricted all analyses to the later era, with alternative era boundaries examined between the 42nd and 60th admissions. Odds ratios (ORs) with 95% confidence intervals (CIs) are reported.

**Propensity-score analysis.** To address confounding by indication, stabilized inverse probability of treatment weights were derived from a propensity model for awake prone positioning that included age, sex, APACHE II score, PaO_2_/FiO_2_ ratio, HFNC FiO_2_, chronic heart failure, and chronic kidney disease. Covariate balance before and after weighting was assessed with standardized mean differences (balance threshold < 0.1), and a weighted outcome model estimated the association with HFNC failure. Propensity-score estimation and weighting were performed within each imputed dataset, and estimates were pooled by Rubin’s rules. Covariates with residual imbalance after weighting (standardized difference ≥ 0.1) were additionally included in the weighted outcome model, and robust (sandwich) standard errors were used.

**Time-to-event and landmark analyses.** Time to intubation was analyzed from ICU admission, which coincided with HFNC initiation in 215 of 222 patients (time zero), with patients not intubated censored at ICU discharge. Although prone positioning was part of the initial HFNC strategy, patients who deteriorated within hours may have had little opportunity to establish prone positioning before intubation, so that the conventional ever/never comparison anchored at time zero may still be affected by immortal-time bias and by confounding related to early deterioration [18,19]. We therefore report the conventional Kaplan–Meier comparison only for reference and base inference on landmark analyses at day 1 (primary) and day 2 (sensitivity), restricting each to patients still at risk at the landmark and estimating Cox hazard ratios (HRs) both unadjusted and adjusted for age, APACHE II score, PaO_2_/FiO_2_ ratio, chronic heart failure, and chronic kidney disease. Day 1 was selected as the primary landmark because it excludes intubations occurring within the first day, when rapid deterioration could preclude the establishment of prone positioning (58 of 149 events, 38.9%), while retaining the majority of events for analysis (91 events); the day-2 landmark excluded a further 26 events and was therefore used as a sensitivity analysis. Cox models were interpreted as cause-specific hazard models, and the proportional-hazards assumption was assessed using scaled Schoenfeld residuals (Grambsch–Therneau test); covariates violating the assumption were handled by stratification. Because patients discharged alive from the ICU or dying in the ICU without intubation could no longer experience the outcome, ICU discharge and ICU death without intubation were additionally treated as competing events. The cumulative incidence of intubation was estimated with the Aalen–Johansen estimator, and subdistribution hazard ratios (sHRs) were estimated with Fine–Gray models in the same landmark cohorts, unadjusted and adjusted for the same covariates. All patients were followed until intubation, ICU discharge, or ICU death, so no administrative censoring occurred. Because prone positioning was not initiated later in the HFNC course, exposure status was established before both landmarks; landmark analyses therefore did not incorporate post-landmark exposure information and primarily served to exclude patients who deteriorated before prone positioning could be established.

## 3. Results

### 3.1. Cohort

**Figure 1:**
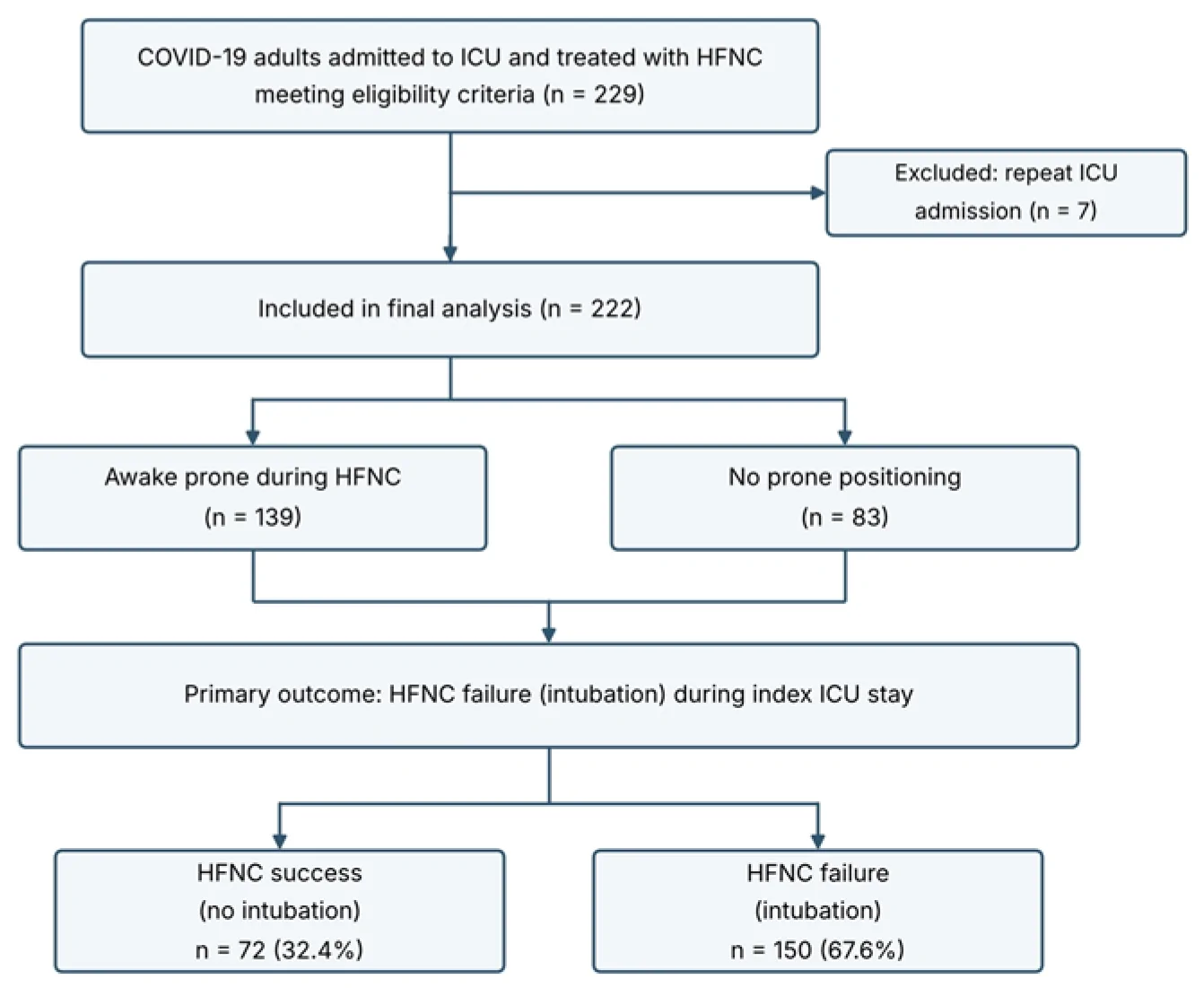
Flow diagram of patient selection and outcomes.

Of 229 patients with COVID-19 who received HFNC and met eligibility criteria, seven were excluded for repeat ICU admissions, leaving 222 patients with classifiable outcomes. HFNC was initiated at ICU admission in 215 patients (96.8%); in the remaining seven, HFNC was started later during the ICU stay, after extubation in four patients admitted on invasive mechanical ventilation and during follow-up in three patients admitted for non-respiratory reasons. Awake prone positioning during HFNC was applied in 139 patients (62.6%) and not applied in 83 (37.4%). The proportion of patients managed with awake prone positioning increased from 38.0% (19/50) in the early era to 69.8% (120/172) in the later era (*p* < 0.001), and the use of COVID-19-specific treatments before ICU admission differed markedly between eras (Supplementary Table S3). HFNC was successful in 72 patients (32.4%) and unsuccessful in 150 (67.6%). The comparatively high failure rate is consistent with the severity of the ICU-referred, predominantly severely hypoxemic population managed during successive pandemic waves (median admission PaO_2_/FiO_2_ ≈ 90). Among patients not intubated, 68 were discharged alive from the ICU and four (1.8% of the cohort) died in the ICU without intubation. The study flow is shown in Figure 1.

### 3.2. Baseline Characteristics and Illness Severity

Baseline demographics, comorbidities, and illness severity are shown in Table 1. Sex, age, and BMI did not differ between groups. Both APACHE II and SOFA scores at ICU admission were higher in the HFNC failure group (both *p* < 0.001). Chronic heart failure and chronic kidney disease were more prevalent among patients with failure (*p* = 0.038 and *p* = 0.009), whereas the Charlson Comorbidity Index was comparable.

### 3.3. Admission Physiology and Laboratory Parameters

At ICU admission, patients with HFNC failure had lower PaO_2_/FiO_2_ ratios (87.5 [70.0–120.0] vs. 100.6 [77.5–150.0]; *p* = 0.011) and higher FiO_2_ requirements (85.0% [60.0–95.0] vs. 67.5% [51.3–85.0]; *p* < 0.001); HFNC flow was similar. Respiratory rate was slightly higher in the successful group (30.0 [26.0–35.0] vs. 28.0 [18.0–35.0] breaths/min; *p* = 0.024); in the absence of a standardized measure of inspiratory effort, this finding should be interpreted cautiously. Inflammatory and cardiac biomarkers—CRP, procalcitonin, D-dimer, pro-B-type natriuretic peptide, and high-sensitivity troponin T—were higher in the failure group (Table 2). These comparisons were exploratory and unadjusted for multiplicity.

**Table 2:** Admission physiology and laboratory parameters according to HFNC outcome.

Variable	Successful	Unsuccessful	*p*
PaO_2_/FiO_2_ ratio	100.6 (77.5–150.0)	87.5 (70.0–120.0)	**0.011**
Respiratory rate, breaths/min	30.0 (26.0–35.0)	28.0 (18.0–35.0)	**0.024**
HFNC flow, L/min	50.0 (50.0–60.0)	50.0 (50.0–60.0)	0.792
HFNC FiO_2_, %	67.5 (51.3–85.0)	85.0 (60.0–95.0)	**<0.001**
CRP, mg/L	88.0 (39.5–161.4)	123.6 (59.9–187.5)	**0.033**
Procalcitonin, ng/mL	0.18 (0.11–0.28)	0.25 (0.12–0.57)	**0.030**
IL-6, pg/mL	129.9 (26.2–442.3)	359.1 (84.5–3664.3)	0.115
Leukocytes, ×10^3^/µL	9.5 (6.9–13.5)	10.2 (7.4–14.9)	0.119
Ferritin, ng/mL	1034.5 (468.3–1788.0)	920.4 (466.2–1615.3)	0.680
D-dimer, ng/mL	1295.0 (787.5–2715.0)	2440 (1222.5–5191.5)	**<0.001**
Glucose, mg/dL	144.8 (118.0–190.0)	147.9 (116.5–185.0)	0.977
AST, U/L	38.0 (27.5–66.0)	35.0 (23.3–55.0)	0.300
ALT, U/L	33.5 (21.5–69.4)	25.0 (15.7–42.3)	**0.013**
Total bilirubin, mg/dL	0.50 (0.37–0.65)	0.43 (0.30–0.61)	0.086
LDH, U/L	433.5 (348.8–570.0)	480.0 (357.0–630.0)	0.307
Lactate, mmol/L	1.6 (1.2–2.1)	1.8 (1.3–2.3)	0.178
Pro-BNP, pg/mL	596.9 (225.2–1946.0)	1045.9 (463.0–4160.0)	**0.005**
hs-Troponin T, ng/L	11.4 (6.8–25.7)	24.1 (11.1–58.8)	**<0.001**
Hemoglobin, g/dL	12.5 (11.0–13.7)	11.6 (10.1–13.1)	**0.042**
Lymphocytes, ×10^3^/µL	0.52 (0.40–0.90)	0.50 (0.30–0.73)	0.239
Platelets, ×10^3^/µL	284.0 (206.0–351.3)	259.5 (196.3–338.5)	0.374
PT, s	13.4 (12.6–14.9)	13.9 (12.9–15.2)	0.137
aPTT, s	28.4 (26.0–32.5)	30.0 (26.8–33.6)	0.059
AT-III, %	89.0 (77.3–98.8)	84.0 (74.0–97.9)	0.309
Fibrinogen, mg/dL	605.0 (481.5–693.8)	613.7 (497.2–693.8)	0.934

Data are median (IQR); groups were compared with the Mann–Whitney U test. Bold *p*-values denote *p* < 0.05 (two-sided). These admission–biomarker comparisons were exploratory and not adjusted for multiple testing; *p*-values are hypothesis-generating. Abbreviations: ALT, alanine aminotransferase; aPTT, activated partial thromboplastin time; AST, aspartate aminotransferase; AT-III, antithrombin III; CRP, C-reactive protein; FiO_2_, fraction of inspired oxygen; HFNC, high-flow nasal cannula; hs-Troponin T, high-sensitivity troponin T; IL-6, interleukin-6; IQR, interquartile range; LDH, lactate dehydrogenase; PaO_2_/FiO_2_, ratio of arterial oxygen partial pressure to fraction of inspired oxygen; pro-BNP, pro-B-type natriuretic peptide; PT, prothrombin time.

### 3.4. Support Trajectories and Outcomes

Patients with HFNC success had longer HFNC duration (6.0 [4.0–9.0] vs. 4.0 [1.0–7.0] days; *p* < 0.001) and were more often managed with awake prone positioning (79.2% vs. 54.7%; *p* < 0.001). Compared with proned patients, patients in whom prone positioning was not established were older (68.3 ± 15.3 vs. 61.3 ± 12.0 years; *p* < 0.001), had a higher comorbidity burden (CCI 5.0 [3.0–7.0] vs. 3.0 [1.0–4.0]; *p* < 0.001), higher APACHE II (17.0 [13.0–21.0] vs. 12.0 [9.0–16.0]; *p* < 0.001) and SOFA scores (5.0 [3.0–8.0] vs. 4.0 [3.0–5.0]; *p* = 0.002), lower mean arterial pressure (89.2 [75.0–100.0] vs. 95.0 [86.7–100.0] mmHg; *p* = 0.017), and higher pro-BNP and high-sensitivity troponin T levels, but higher PaO_2_/FiO_2_ ratios (101.7 [74.2–149.5] vs. 89.6 [70.9–112.8]; *p* = 0.017); they were also less often treated with corticosteroids before ICU admission (48.2% vs. 71.9%; *p* < 0.001) (Supplementary Table S3). Cumulative documented prone days were greater in the success group (5.0 [3.0–7.0] vs. 3.0 [1.0–7.0]; *p* = 0.015); this descriptive difference reflects the clinical trajectory and time on HFNC and is not interpreted causally. Patients with failure more often required vasopressor/inotropic support (78.0% vs. 6.9%), RRT (31.3% vs. 4.2%), and sedation (32.0% vs. 18.1%), had longer ICU stay (11.0 [7.0–18.0] vs. 7.0 [5.0–10.8] days), and had higher ICU mortality (66.7% vs. 5.6%; all *p* ≤ 0.029). Hospital length of stay did not differ (Table 3). SOFA scores during follow-up were higher in the failure group (maximum: 9.0 [7.0–13.5] vs. 4.0 [3.0–5.3]; day 6: 5.0 [4.0–8.0] vs. 2.0 [2.0–4.0]; both *p* < 0.001). Day-6 SOFA was available in 116 patients (42 of 72 successful and 74 of 150 unsuccessful), as availability depended on remaining in the ICU; these values reflect the subsequent clinical course rather than baseline status and are shown descriptively.

### 3.5. Multivariable and Propensity-Score Analyses

In the multivariable logistic model with multiple imputation (*n* = 222), awake prone positioning was associated with lower HFNC failure (OR 0.23; 95% CI 0.10–0.53; *p* < 0.001), whereas higher APACHE II score (OR 1.09 per point; 95% CI 1.02–1.17; *p* = 0.012), higher HFNC FiO_2_ (OR 1.41 per 10% increment; 95% CI 1.15–1.73; *p* < 0.001), and lower admission PaO_2_/FiO_2_ ratio (OR 0.9948 per unit; 95% CI 0.9900–0.9997; *p* = 0.037) were independently associated with failure. HFNC FiO_2_ was modeled per 10% increment because modeling it as a single 0–1 unit yielded an unstable estimate. In the SOFA-adjusted sensitivity model, awake prone positioning remained associated with lower failure (OR 0.24; 95% CI 0.10–0.57; *p* = 0.001), and SOFA (OR 1.30 per point; 95% CI 1.06–1.59; *p* = 0.013) and HFNC FiO_2_ (OR 1.37 per 10% increment; 95% CI 1.11–1.69; *p* = 0.003) were independently associated with failure (Table 4). Complete-case analysis yielded consistent estimates for awake prone positioning (APACHE II model: *n* = 118, 78 events; OR 0.24; 95% CI 0.08–0.74; *p* = 0.013; SOFA model: *n* = 106, 65 events; OR 0.27; 95% CI 0.09–0.84; *p* = 0.024) (Supplementary Table S2). In the stabilized IPTW analysis, all post-weighting standardized differences were <0.1 except for age (0.19) and APACHE II score (0.13), which were therefore additionally included in the weighted outcome model; awake prone positioning remained associated with lower failure (OR 0.22; 95% CI 0.10–0.47; *p* < 0.001). In the sensitivity analysis restricted to the 215 patients in whom HFNC was initiated at ICU admission, results were materially unchanged (OR 0.25; 95% CI 0.11–0.56 in the APACHE II model and OR 0.26; 95% CI 0.11–0.60 in the SOFA model; IPTW OR 0.24; 95% CI 0.11–0.52) (Supplementary Table S4). In the extended model additionally adjusted for mean arterial pressure, heart rate, Charlson Comorbidity Index, and corticosteroid and tocilizumab treatment before ICU admission, the association of awake prone positioning with lower HFNC failure was unchanged (OR 0.20; 95% CI 0.08–0.49; *p* < 0.001). Additional adjustment for calendar time did not attenuate the association (OR 0.18; 95% CI 0.07–0.45; IPTW OR 0.21; 95% CI 0.09–0.46), nor did further adjustment for corticosteroid, tocilizumab, and anakinra treatment (OR 0.17; 95% CI 0.07–0.44). When analyses were restricted to the later era (*n* = 172; 119 events), the association persisted (multivariable OR 0.24; 95% CI 0.08–0.73; *p* = 0.011; IPTW OR 0.22; 95% CI 0.08–0.59; *p* = 0.003); across alternative era boundaries between the 42nd and 60th admissions, multivariable ORs ranged from 0.23 to 0.33 (Supplementary Table S5).

### 3.6. Time-to-Event and Landmark Analyses

**Figure 2:**
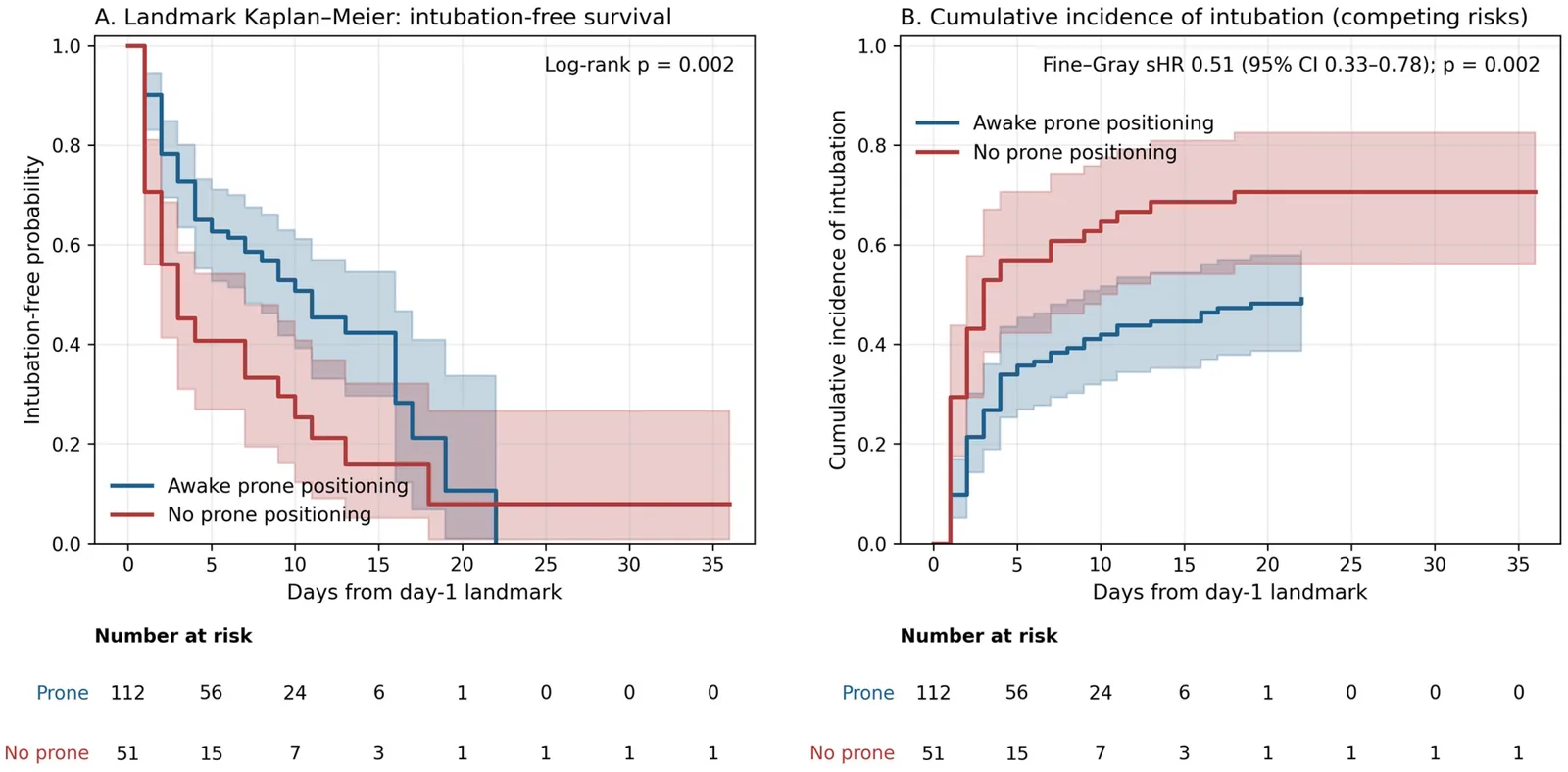
Time to intubation from a day-1 landmark, stratified by awake prone positioning during HFNC. (**A**) Landmark Kaplan–Meier curves for intubation-free survival (log-rank test). (**B**) Cumulative incidence of intubation estimated with the Aalen–Johansen estimator, treating ICU discharge and ICU death without intubation as competing events (Fine–Gray model). Shaded areas indicate 95% confidence intervals; numbers at risk are shown below each panel.

The conventional Kaplan–Meier comparison anchored at time zero showed a lower observed incidence of intubation among patients undergoing awake prone positioning (log-rank *p* < 0.001). This estimate is likely influenced by early deterioration: among patients who ultimately failed, the proportion who had been proned was 45% for those intubated within 2 days and 68% for those intubated later, consistent with patients who deteriorated rapidly being less able to establish or tolerate prone positioning. Consistent with proning being part of the initial HFNC strategy, even among patients intubated on the day of HFNC initiation, 20 of 31 (64.5%) had already been proned. Landmark analyses excluding early events were therefore used for inference. After a day-1 landmark (*n* = 163; 91 events), the association attenuated but remained significant (HR 0.52; 95% CI 0.34–0.79; *p* = 0.002; adjusted HR 0.42; 95% CI 0.26–0.69; *p* < 0.001; Figure 2A). At a day-2 landmark (*n* = 133; 65 events) the unadjusted association was non-significant (HR 0.66; 95% CI 0.39–1.11; *p* = 0.118) but became significant after adjustment for illness severity and comorbidity (HR 0.47; 95% CI 0.25–0.88; *p* = 0.019), in which model APACHE II (HR 1.06 per point; *p* = 0.026) and PaO_2_/FiO_2_ (HR 0.994 per unit; *p* = 0.008) remained independent correlates (Table 5). Landmark estimates were similar in the 215 patients in whom HFNC was initiated at ICU admission (day 1: HR 0.50; 95% CI 0.32–0.77; adjusted HR 0.42; 95% CI 0.26–0.69; day 2: adjusted HR 0.47; 95% CI 0.25–0.88) (Supplementary Table S4). After additional adjustment for calendar time, adjusted landmark estimates were similar (day 1: HR 0.44; 95% CI 0.26–0.74; day 2: HR 0.47; 95% CI 0.25–0.90). In the later era, the adjusted HRs were 0.48 (95% CI 0.27–0.87; *p* = 0.016) on day 1 and 0.48 (95% CI 0.23–0.97; *p* = 0.041) on day 2, and the adjusted day-1 HR ranged from 0.48 to 0.53 across alternative era boundaries (Supplementary Table S5). The proportional-hazards assumption was not violated for awake prone positioning in any landmark model (Grambsch–Therneau *p* ≥ 0.074); chronic heart failure violated the assumption in the adjusted models (*p* = 0.030 at day 1 and *p* = 0.033 at day 2), and stratification by chronic heart failure yielded similar estimates (day 1: HR 0.43; 95% CI 0.26–0.70; day 2: HR 0.47; 95% CI 0.25–0.88). In the competing-risk analysis, the cumulative incidence of intubation 14 days after the day-1 landmark was 45% (95% CI 35–54%) with awake prone positioning and 69% (95% CI 54–81%) without (Figure 2B). Fine–Gray estimates were consistent with the cause-specific analyses at day 1 (sHR 0.51; 95% CI 0.33–0.78; *p* = 0.002; adjusted sHR 0.51; 95% CI 0.31–0.84; *p* = 0.008); at day 2, they were directionally consistent but not statistically significant (sHR 0.61; 95% CI 0.36–1.02; *p* = 0.059; adjusted sHR 0.57; 95% CI 0.31–1.06; *p* = 0.074) (Table 5).

## 4. Discussion

In this single-center ICU cohort of adults with COVID-19-associated acute hypoxemic respiratory failure, patients undergoing awake prone positioning during HFNC had a lower observed incidence of intubation, whereas greater illness severity and more severe hypoxemia at ICU admission were independently associated with HFNC failure. Two features distinguish our analysis from prior observational reports. First, we modeled illness severity explicitly and found that APACHE II and, in sensitivity analysis, SOFA retained independent prognostic values after adjustment, and that oxygenation variables were also independently associated with failure (HFNC FiO_2_ in both models and PaO_2_/FiO_2_ in the APACHE II model), whereas inflammatory biomarkers did not materially alter the proning–failure association. Second, because prone positioning was part of the initial HFNC strategy, exposure was established at baseline; nevertheless, we explicitly examined time-related bias: the crude survival analysis overstated the difference in intubation between proned and non-proned patients, and after landmark analysis the association was more modest and sensitive to landmark timing—significant at day 1 and, at day 2, only after adjustment for severity. This nuance is easily missed when early deterioration and exposure timing are not considered in observational analyses [18,19].

These findings should be read alongside the randomized evidence. The Ehrmann meta-trial demonstrated that awake prone positioning reduced treatment failure and intubation in HFNC-supported COVID-19 patients [8], and meta-analysis supports a reduction in intubation concentrated in the HFNC subgroup [9]. Conversely, the COVI-PRONE trial found no effect on intubation [10], and heterogeneity in adherence, dose, timing, and selection has repeatedly been implicated in discordant results [12,13]. Although our institutional practice targeted the longest tolerated sessions, continued day and night, daily prone duration was not recorded for individual patients, which precludes direct comparison with the exposure achieved in the randomized trials. Our observational estimate is directionally consistent with the positive trials but was attenuated and specification-dependent after bias mitigation; because of residual confounding, it cannot establish whether awake prone positioning reduces the need for intubation. Importantly, patients in whom prone positioning could not be established were older and had more comorbidities, higher APACHE II and SOFA scores, and lower arterial pressure despite less severe hypoxemia, indicating that the ability to tolerate proning is itself a marker of lower risk. Although this was addressed by multivariable adjustment and IPTW, residual confounding by tolerance cannot be excluded and may account for part of the observed association. Temporal changes in management are a further potential confounder, as awake proning became routine in our unit at the same time as corticosteroids replaced earlier therapies; however, the association was not attenuated by adjustment for calendar time and COVID-19-specific treatments and persisted when analyses were restricted to the era of routine proning.

The association of global illness severity with failure aligns with evidence that outcomes in ARDS and COVID-19 often reflect downstream systemic organ dysfunction rather than refractory hypoxemia alone. In ARDS, death frequently follows progression to multiorgan failure despite stabilized gas exchange [22], and large COVID-19 cohorts link higher admission severity scores to intubation and death [15,16]. Our adjusted analyses reinforce this: illness severity retained independent value alongside oxygenation variables, suggesting that reliance on oxygenation metrics alone may underestimate failure risk in patients with evolving systemic disease.

HFNC complements awake proning through delivery of stable high FiO_2_, dead-space washout, and low-level positive airway pressure, collectively reducing the work of breathing [1,2]. Prone positioning reduces mortality in intubated moderate-to-severe ARDS [23], and awake proning extends recruitment benefits to non-intubated patients, with early observational cohorts reporting short-term oxygenation gains, particularly when combined with HFNC [6,7,24,25]. Physiological considerations suggest awake proning may attenuate patient self-inflicted lung injury in tachypneic patients [26], and adjuncts such as dexmedetomidine have been used to improve tolerance and prolong prone sessions [27]. Although early pandemic guidance discouraged HFNC over aerosolization concerns, subsequent work indicated that HFNC does not substantially increase droplet dispersion versus conventional devices, particularly with a surgical mask over the cannula [28,29], supporting sustained use across successive waves.

Systemic inflammation and thromboinflammation correlated with HFNC trajectories: patients who failed had higher admission CRP, procalcitonin, D-dimer, pro-BNP, and troponin T, consistent with the hyperinflammatory, prothrombotic phenotype of severe COVID-19 [16]. However, neither D-dimer nor CRP materially altered the proning–failure association when added to the models, so their value is best regarded as descriptive of a higher-risk phenotype rather than as independent, modifiable predictors in this cohort. Comorbidity burden further shaped prognosis: chronic heart failure and chronic kidney disease were more prevalent among patients with failure, consistent with the prior COVID-19 and respiratory-infection literature [16,30,31,32], plausibly reflecting limited cardiac and renal reserve.

### 4.1. Clinical Implications

Our findings support a pragmatic, severity-informed approach to HFNC in COVID-19-associated acute hypoxemic respiratory failure. Early assessment of global illness severity (APACHE II or SOFA) alongside oxygenation metrics may help identify patients at a higher risk of failure who warrant closer monitoring and readiness for timely escalation; such assessment should complement, rather than replace, serial bedside evaluation with validated tools such as the ROX index [5]. The lower observed incidence of intubation among patients undergoing awake prone positioning was directionally consistent across imputed multivariable, IPTW, landmark, and competing-risk analyses; however, given residual confounding by tolerance and clinical stability, these data do not establish a treatment effect, and the role of awake proning should be defined by randomized evidence.

### 4.2. Strengths and Limitations

Strengths include a relatively large single-center cohort with granular physiological, laboratory, and treatment data collected across multiple pandemic waves under a consistent institutional approach, and the use of complementary analytic strategies—multiple imputation, propensity-score weighting, and landmark time-to-event analysis—that directly address the principal biases threatening observational inference in this setting.

Several limitations remain. First, the retrospective single-center design permits residual and unmeasured confounding despite adjustment and weighting; although prone positioning was routinely considered in all patients, it could be established only in those in whom it was clinically feasible and tolerated, so more cooperative, more stable, or systemically less severely ill patients were more likely to be proned, and the association should not be read as a causal treatment effect. Neurological status, agitation or delirium, work of breathing, and the ability to cooperate, which determined eligibility for proning in clinical practice, were not recorded as structured variables and could not be adjusted for, although additional adjustment for hemodynamic variables, comorbidity burden, and pre-ICU treatments did not change the results. Moreover, because clinical status evolves during the HFNC course and daily clinical data were not recorded, time-varying confounding could not be addressed with methods such as marginal structural models, and the residual time-related bias cannot be fully excluded. Second, missingness in BMI, respiratory rate, and SOFA (33–36%) was addressed by multiple imputation, but imputation cannot recover information absent from the records. Third, the exact time of the first prone session and the reasons why prone positioning could not be established were not recorded for individual patients; the timing of exposure is based on the institutional practice of considering and initiating proning as part of the initial HFNC strategy. Similarly, session-level data (duration and number of sessions per day, reasons for termination, and adherence) were not recorded for individual patients, so actual daily prone exposure could not be quantified. Likewise, although arterial blood gases were obtained routinely four times daily, the timing of each sample relative to individual prone sessions and to intubation was not consistently documented, and SpO_2_ was not abstracted into the study dataset; serial physiological responses to HFNC and awake prone positioning (before and after proning and immediately before intubation) therefore could not be reliably reconstructed. Dose–response could not be assessed, because cumulative prone duration reflected the clinical trajectory rather than treatment dose. Fourth, the study spanned multiple pandemic phases with evolving variants, vaccination coverage, and adjunctive therapies; calendar time was represented by admission order rather than by the pandemic wave, and vaccination status and viral variant were not recorded. Estimates restricted to the later era were less precise because of the smaller sample size. Fifth, decisions on HFNC initiation, sedation, and escalation were clinician-driven and may reflect unmeasured factors such as staffing or bedside judgment. In particular, no standardized, protocol-based intubation criteria were applied; because the threshold for intubation was clinical rather than numeric, variability between clinicians and its potential influence on the primary outcome cannot be excluded, and the extent of delayed intubation could not be quantified. Sixth, in seven patients HFNC was started after ICU admission, so their admission covariates may not reflect their condition at HFNC initiation; however, excluding these patients did not materially change the results. Seventh, the ROX index could not be calculated, because SpO_2_ was not recorded in the study dataset, respiratory rate was missing in 33% of patients, and the ROX index has been validated at 2, 6, and 12 h after HFNC initiation, whereas our physiological variables were obtained at ICU admission; our severity-based findings therefore could not be compared directly with this established bedside tool. Finally, ICU discharge precluded subsequent intubation during the index stay; competing-risk analyses yielded consistent estimates at the day-1 landmark, whereas the day-2 subdistribution estimates were directionally consistent but not statistically significant.

## 5. Conclusions

In critically ill patients with COVID-19-associated acute hypoxemic respiratory failure managed with HFNC, patients undergoing awake prone positioning had a lower observed incidence of intubation, whereas greater illness severity and more severe hypoxemia at ICU admission were independently associated with failure. This difference was directionally consistent across imputed multivariable, propensity-score, and landmark analyses but more modest than crude analyses implied and sensitive to landmark timing, underscoring the need for bias-aware interpretation of observational data. These findings support severity-informed monitoring and timely escalation during HFNC. Causality cannot be inferred because of the observational design and residual confounding, particularly confounding related to tolerance of proning, and prospective studies should clarify optimal patient selection, timing, and implementation.

## Figures and Tables

**Table 1:** Baseline demographics, comorbidities, and illness severity at ICU entry.

Variable	Overall	Successful	Unsuccessful	*p*
Sex, male	148 (66.7)	51 (70.8)	97 (64.7)	0.362
Age, years	63.9 ± 13.7	61.9 ± 14.3	64.9 ± 13.3	0.143
BMI, kg/m^2^	25.9 (24.5–28.4)	25.4 (24.5–27.6)	25.9 (24.5–29.4)	0.343
APACHE II at ICU entry	14.0 (10.0–18.0)	11.0 (8.0–16.0)	14.0 (11.0–19.0)	**<0.001**
SOFA at ICU entry	4.0 (3.0–6.0)	4.0 (3.0–4.5)	4.0 (3.0–7.0)	**<0.001**
Cerebrovascular disease	15 (6.8)	4 (5.6)	11 (7.3)	0.779
Chronic heart failure	80 (36.0)	19 (26.4)	61 (40.7)	**0.038**
Chronic kidney disease	32 (14.5)	4 (5.6)	28 (18.7)	**0.009**
Chronic liver disease	4 (1.8)	1 (1.4)	3 (2.0)	1.000
Chronic pulmonary disease	48 (21.6)	16 (22.2)	32 (21.3)	0.880
Diabetes mellitus	80 (36.0)	24 (33.3)	56 (37.3)	0.561
Hypertension	116 (52.3)	33 (45.8)	83 (55.3)	0.185
Malignancy	24 (10.9)	4 (5.6)	20 (13.4)	0.078
Transplant recipient	9 (4.1)	1 (1.4)	8 (5.3)	0.277
Charlson Comorbidity Index	3.0 (2.0–5.0)	3.0 (1.0–4.0)	4.0 (2.0–5.0)	0.118

Data are *n* (%) for categorical variables and mean ± SD or median (IQR) for continuous variables. Categorical variables were compared with the χ^2^ or Fisher exact test and continuous variables with the Student *t*-test or Mann–Whitney U test, as appropriate. Bold *p*-values denote *p* < 0.05 (two-sided). Successful = HFNC maintained without intubation; unsuccessful = intubated during the index ICU stay. Abbreviations: APACHE II, Acute Physiology and Chronic Health Evaluation II; BMI, body mass index; ICU, intensive care unit; IQR, interquartile range; SD, standard deviation; SOFA, Sequential Organ Failure Assessment.

**Table 3:** Respiratory/organ-support trajectories and outcomes.

Variable	Overall	Successful	Unsuccessful	*p*
HFNC duration, days	5.0 (2.0–8.0)	6.0 (4.0–9.0)	4.0 (1.0–7.0)	**<0.001**
Awake prone during HFNC	139 (62.6)	57 (79.2)	82 (54.7)	**<0.001**
Cumulative prone days	4.0 (2.0–7.0)	5.0 (3.0–7.0)	3.0 (1.0–7.0)	**0.015**
Vasopressor/inotrope	122 (55.0)	5 (6.9)	117 (78.0)	**<0.001**
Renal replacement therapy	50 (22.5)	3 (4.2)	47 (31.3)	**<0.001**
Cytokine adsorption	5 (2.3)	1 (1.4)	4 (2.7)	1.000
Sedation	61 (27.5)	13 (18.1)	48 (32.0)	**0.029**
ECMO	7 (3.2)	0 (0)	7 (4.7)	0.099
ICU length of stay, days	10.0 (6.0–15.0)	7.0 (5.0–10.8)	11.0 (7.0–18.0)	**<0.001**
Hospital length of stay, days	19.0 (13.0–28.0)	19.0 (13.0–24.0)	19.0 (13.0–29.0)	0.400
ICU mortality	104 (46.8)	4 (5.6)	100 (66.7)	**<0.001**
SOFA, maximum †	7.0 (4.0–10.0)	4.0 (3.0–5.3)	9.0 (7.0–13.5)	**<0.001**
SOFA, day 6 †	4.0 (2.3–7.0)	2.0 (2.0–4.0)	5.0 (4.0–8.0)	**<0.001**

Data are median (IQR) for durations and *n* (%) for proportions. Continuous variables were compared with the Mann–Whitney U test and categorical variables with the χ^2^ or Fisher exact test. Bold *p*-values denote *p* < 0.05 (two-sided). Cumulative prone days are shown descriptively and were not interpreted causally because they depend on time remaining on HFNC. † Follow-up variables reflecting the subsequent clinical course rather than baseline status. Maximum SOFA during the ICU stay was available in 139 patients (54 successful, 85 unsuccessful) and SOFA on ICU day 6 in 116 patients (42 successful, 74 unsuccessful); availability on day 6 depended on remaining in the ICU, so these values are subject to selection and are shown descriptively. Abbreviations: ECMO, extracorporeal membrane oxygenation; HFNC, high-flow nasal cannula; ICU, intensive care unit; IQR, interquartile range; SOFA, Sequential Organ Failure Assessment.

**Table 4:** Multivariable logistic regression for HFNC failure (multiple imputation, m = 25; FiO_2_ per 10% increment).

Parameter	APACHE II Model, OR (95% CI)	*p*	SOFA Model, OR (95% CI)	*p*
Awake prone positioning (yes vs. no)	0.23 (0.10–0.53)	**<0.001**	0.24 (0.10–0.57)	**0.001**
Age, years	0.98 (0.95–1.01)	0.242	1.00 (0.97–1.02)	0.731
Sex (male vs. female)	1.05 (0.49–2.25)	0.904	1.01 (0.47–2.17)	0.971
BMI, kg/m^2^	1.05 (0.94–1.17)	0.360	1.04 (0.93–1.16)	0.520
APACHE II (per point)	1.09 (1.02–1.17)	**0.012**	—	—
SOFA (per point)	—	—	1.30 (1.06–1.59)	**0.013**
PaO_2_/FiO_2_ ratio (per unit)	0.9948 (0.9900–0.9997)	**0.037**	0.9964 (0.9916–1.0012)	0.138
HFNC FiO_2_ (per 10%)	1.41 (1.15–1.73)	**<0.001**	1.37 (1.11–1.69)	**0.003**
Respiratory rate	1.00 (0.95–1.05)	0.871	1.01 (0.96–1.06)	0.757
Chronic heart failure	1.78 (0.81–3.92)	0.153	1.50 (0.67–3.38)	0.328
Chronic kidney disease	1.71 (0.49–6.03)	0.403	1.51 (0.39–5.77)	0.550

Values are odds ratios (95% CI) from multivariable binary logistic regression for HFNC failure (intubation). Missing covariate data (BMI, respiratory rate, SOFA: 33–36%; others ≤ 3.2%) were handled by multiple imputation using chained equations with predictive mean matching (m = 25 imputations), with estimates pooled by Rubin’s rules; the analytic sample is the full cohort (*n* = 222; 150 events). Covariates were pre-specified on clinical grounds. HFNC FiO_2_ is expressed per 10% absolute increment. The APACHE II and SOFA models are otherwise identical and differ only in the severity index entered. The reference category for awake prone positioning is “no prone positioning.” Bold *p*-values denote *p* < 0.05 (two-sided). Abbreviations: APACHE II, Acute Physiology and Chronic Health Evaluation II; BMI, body mass index; CI, confidence interval; FiO_2_, fraction of inspired oxygen; HFNC, high-flow nasal cannula; OR, odds ratio; PaO_2_/FiO_2_, ratio of arterial oxygen partial pressure to fraction of inspired oxygen; SOFA, Sequential Organ Failure Assessment.

**Table 5:** Landmark Cox analyses for time to intubation by awake prone positioning.

Analysis	*n* (Events)	HR (95% CI)	*p*
Conventional KM (anchored at time zero) †	221 (149)	—	<0.001 (log-rank)
Day-1 landmark, unadjusted	163 (91)	0.52 (0.34–0.79)	**0.002**
Day-1 landmark, adjusted ‡	158 (89)	0.42 (0.26–0.69)	**<0.001**
Day-2 landmark, unadjusted	133 (65)	0.66 (0.39–1.11)	0.118
Day-2 landmark, adjusted ‡	129 (64)	0.47 (0.25–0.88)	**0.019**
Competing-risk analysis (Fine–Gray) §			
Day-1 landmark, unadjusted	163 (91)	0.51 (0.33–0.78)	**0.002**
Day-1 landmark, adjusted ‡	158 (89)	0.51 (0.31–0.84)	**0.008**
Day-2 landmark, unadjusted	133 (65)	0.61 (0.36–1.02)	0.059
Day-2 landmark, adjusted ‡	129 (64)	0.57 (0.31–1.06)	0.074

Values are cause-specific hazard ratios (95% CI) for time to intubation from Cox proportional-hazards models, unless otherwise indicated. Time zero was ICU admission, which coincided with HFNC initiation in 215 of 222 patients; patients not intubated were censored at ICU discharge. Landmark analyses were restricted to patients still at risk (neither intubated nor discharged) on the landmark day, with the clock reset to the landmark; awake prone positioning was entered as a fixed exposure, established during the initial HFNC period and therefore before each landmark. Bold *p*-values denote *p* < 0.05 (two-sided). † Conventional Kaplan–Meier comparison anchored at time zero (log-rank test); reported for reference only and potentially influenced by early deterioration before prone positioning could be established. One patient, admitted on invasive mechanical ventilation and started on HFNC after extubation, whose recorded HFNC FiO_2_ was 0 and was considered implausible (Section 2.6), was not included in the time-to-event analyses (*n* = 221; 149 events). ‡ Adjusted for age, APACHE II score, PaO_2_/FiO_2_ ratio, chronic heart failure, and chronic kidney disease. § Subdistribution hazard ratios, with ICU discharge and ICU death without intubation as competing events. The proportional-hazards assumption for awake prone positioning was not violated in any Cox model (Grambsch–Therneau test, *p* ≥ 0.074). Abbreviations: APACHE II, Acute Physiology and Chronic Health Evaluation II; CI, confidence interval; HFNC, high-flow nasal cannula; HR, hazard ratio; ICU, intensive care unit; KM, Kaplan–Meier; PaO_2_/FiO_2_, ratio of arterial oxygen partial pressure to fraction of inspired oxygen; sHR, subdistribution hazard ratio.

## Data Availability

The de-identified clinical dataset is available from the corresponding author upon reasonable request.

## Supplementary Materials

The following supporting information can be downloaded at: https://www.mdpi.com/article/10.3390/medicina62101984/s1, Figure S1: Convergence diagnostics for multiple imputation; Table S1: Missing data for each variable, overall and according to outcome and awake prone positioning; Table S2: Complete-case multivariable logistic regression for HFNC failure; Table S3: Baseline characteristics according to awake prone positioning during HFNC; Table S4: Sensitivity analyses restricted to patients in whom HFNC was initiated at ICU admission; Table S5: Sensitivity analyses accounting for calendar time and COVID-19-specific treatments.

## Abbreviations

The following abbreviations are used in this manuscript:

ALT	alanine aminotransferase
APACHE II	Acute Physiology and Chronic Health Evaluation II
ARDS	acute respiratory distress syndrome
AST	aspartate aminotransferase
AT-III	antithrombin III
BMI	body mass index
CCI	Charlson Comorbidity Index
CI	confidence interval
COVID-19	coronavirus disease 2019
CPAP	continuous positive airway pressure
CRP	C-reactive protein
ECMO	extracorporeal membrane oxygenation
FiO_2_	fraction of inspired oxygen
HFNC	high-flow nasal cannula
HR	hazard ratio
ICU	intensive care unit
IPTW	inverse probability of treatment weighting
IQR	interquartile range
IL-6	interleukin-6
LDH	lactate dehydrogenase
OR	odds ratio
PaO_2_	partial pressure of arterial oxygen
Pro-BNP	pro-B-type natriuretic peptide
ROX	respiratory rate–oxygenation index
RRT	renal replacement therapy
sHR	subdistribution hazard ratio
SMD	standardized mean difference
SOFA	Sequential Organ Failure Assessment
SpO_2_	peripheral oxygen saturation
STROBE	Strengthening the Reporting of Observational Studies in Epidemiology
