# Supplementary material for: High-Flow Nasal Cannula Outcomes in COVID-19-Associated Acute Hypoxemic Respiratory Failure: Illness Severity, Awake Prone Positioning, and Risk of Intubation in a Single-Center ICU Cohort
Source: Medicina (Kaunas). 2026 Oct 14;62(10):1984. doi: 10.3390/medicina62101984 (PMC13707056; doi:10.3390/medicina62101984)
Supplement: Supplementary file 1 [file medicina-62-01984-s001.zip › medicina-4573609-supplementary.pdf]

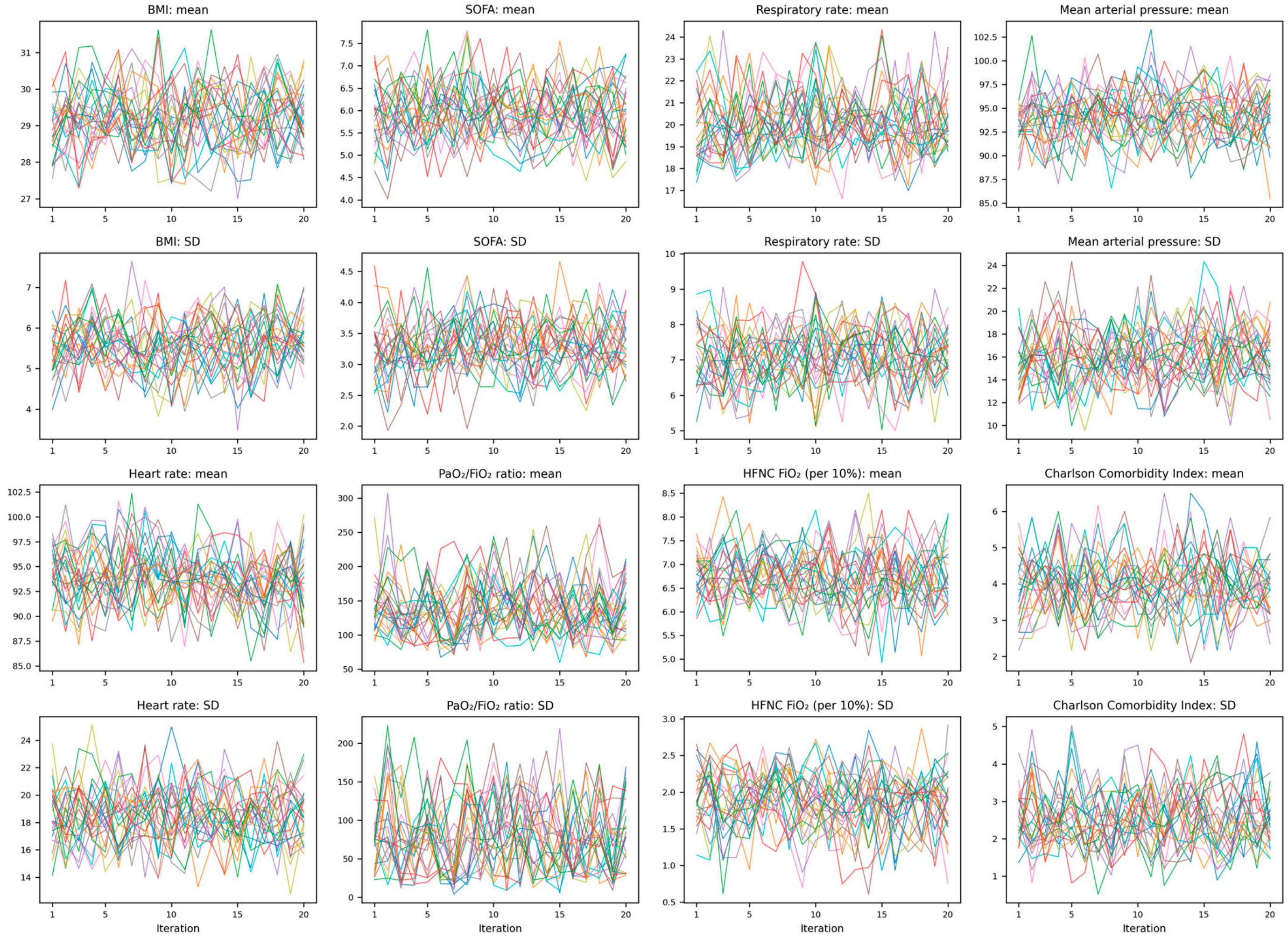

Supplementary Figure S1. Convergence diagnostics for multiple imputation. Trace plots of the mean and standard deviation of imputed values across 20 iterations for each variable with missing data; each line represents one of the 25 independent imputation chains. APACHE II (one missing value) is not shown. Abbreviations: BMI, body mass index; FiO_2, fraction of inspired oxygen; HFNC, high-flow nasal cannula; PaO_2/FiO_2, ratio of arterial oxygen partial pressure to fraction of inspired oxygen; SOFA, Sequential Organ Failure Assessment.

Table S1. Missing data for each variable, overall and according to outcome and awake prone positioning.

Variable	Overall (n = 222)	Successful (n = 72)	Unsuccessful (n = 150)	Prone (n = 139)	No prone (n = 83)
Model covariates					
BMI	75 (33.8)	21 (29.2)	54 (36.0)	53 (38.1)	22 (26.5)
APACHE II	1 (0.5)	1 (1.4)	0 (0.0)	1 (0.7)	0 (0.0)
SOFA at ICU entry	79 (35.6)	15 (20.8)	64 (42.7)	53 (38.1)	26 (31.3)
PaO_2/FiO_2 ratio	4 (1.8)	2 (2.8)	2 (1.3)	1 (0.7)	3 (3.6)
HFNC FiO_2	7 (3.2)	0 (0.0)	7 (4.7)	0 (0.0)	7 (8.4)
Respiratory rate	74 (33.3)	30 (41.7)	44 (29.3)	52 (37.4)	22 (26.5)
Other baseline variables					
Charlson Comorbidity Index	6 (2.7)	2 (2.8)	4 (2.7)	3 (2.2)	3 (3.6)
HFNC flow	6 (2.7)	0 (0.0)	6 (4.0)	0 (0.0)	6 (7.2)
Heart rate	47 (21.2)	7 (9.7)	40 (26.7)	33 (23.7)	14 (16.9)
Systolic blood pressure	43 (19.4)	7 (9.7)	36 (24.0)	32 (23.0)	11 (13.3)

Variable	Overall (n = 222)	Successful (n = 72)	Unsuccessful (n = 150)	Prone (n = 139)	No prone (n = 83)
Mean arterial pressure	44 (19.8)	7 (9.7)	37 (24.7)	33 (23.7)	11 (13.3)
Lactate	72 (32.4)	18 (25.0)	54 (36.0)	48 (34.5)	24 (28.9)
Laboratory parameters					
CRP	1 (0.5)	0 (0.0)	1 (0.7)	0 (0.0)	1 (1.2)
Procalcitonin	5 (2.3)	1 (1.4)	4 (2.7)	4 (2.9)	1 (1.2)
IL-6	178 (80.2)	54 (75.0)	124 (82.7)	120 (86.3)	58 (69.9)
Leukocytes	3 (1.4)	0 (0.0)	3 (2.0)	3 (2.2)	0 (0.0)
Ferritin	2 (0.9)	0 (0.0)	2 (1.3)	1 (0.7)	1 (1.2)
D-dimer	2 (0.9)	0 (0.0)	2 (1.3)	1 (0.7)	1 (1.2)
Blood glucose	4 (1.8)	1 (1.4)	3 (2.0)	2 (1.4)	2 (2.4)
AST	8 (3.6)	5 (6.9)	3 (2.0)	5 (3.6)	3 (3.6)
ALT	3 (1.4)	2 (2.8)	1 (0.7)	3 (2.2)	0 (0.0)
Total bilirubin	2 (0.9)	0 (0.0)	2 (1.3)	2 (1.4)	0 (0.0)
LDH	17 (7.7)	6 (8.3)	11 (7.3)	9 (6.5)	8 (9.6)
Pro-BNP	4 (1.8)	0 (0.0)	4 (2.7)	1 (0.7)	3 (3.6)
hs-Troponin T	8 (3.6)	2 (2.8)	6 (4.0)	7 (5.0)	1 (1.2)
Hemoglobin	4 (1.8)	1 (1.4)	3 (2.0)	3 (2.2)	1 (1.2)
Lymphocytes	5 (2.3)	1 (1.4)	4 (2.7)	4 (2.9)	1 (1.2)
Platelets	2 (0.9)	0 (0.0)	2 (1.3)	2 (1.4)	0 (0.0)
PT	1 (0.5)	1 (1.4)	0 (0.0)	1 (0.7)	0 (0.0)
aPTT	2 (0.9)	0 (0.0)	2 (1.3)	2 (1.4)	0 (0.0)
AT-III	8 (3.6)	3 (4.2)	5 (3.3)	2 (1.4)	6 (7.2)
Fibrinogen	2 (0.9)	0 (0.0)	2 (1.3)	1 (0.7)	1 (1.2)

Values are number (%) of patients with missing data. No data were missing for age, sex, comorbidities, awake prone positioning, or outcome. One recorded HFNC FiO_2 value of 0 was considered implausible and counted as missing. IL-6 was not included in any model. Abbreviations: ALT, alanine aminotransferase; APACHE II, Acute Physiology and Chronic Health Evaluation II; aPTT, activated partial thromboplastin time; AST, aspartate aminotransferase; AT-III, antithrombin III; BMI, body mass index; CRP, C-reactive protein; FiO_2, fraction of inspired oxygen; HFNC, high-flow nasal cannula; hs-Troponin T, high-sensitivity troponin T; IL-6, interleukin-6; LDH, lactate dehydrogenase; PaO_2/FiO_2, ratio of arterial oxygen partial pressure to fraction of inspired oxygen; Pro-BNP, pro-B-type natriuretic peptide; PT, prothrombin time; SOFA, Sequential Organ Failure Assessment.

Table S2. Complete-case multivariable logistic regression for HFNC failure.

Parameter	APACHE II model, OR (95% CI)	*p*	SOFA model, OR (95% CI)	*p*
Awake prone positioning (yes vs. no)	0.24 (0.08–0.74)	**0.013**	0.27 (0.09–0.84)	**0.024**
Age, years	0.99 (0.95–1.03)	0.525	0.99 (0.95–1.03)	0.631
Sex (male vs. female)	1.20 (0.44–3.31)	0.725	1.36 (0.49–3.80)	0.561
BMI, kg/m◎	1.15 (0.95–1.38)	0.148	1.15 (0.92–1.45)	0.227
APACHE II (per point)	1.09 (1.01–1.19)	**0.034**	—	—
SOFA (per point)	—	—	1.35 (1.02–1.78)	**0.036**
PaO_2/FiO_2 ratio (per unit)	0.993 (0.984–1.003)	0.152	0.994 (0.985–1.003)	0.192
HFNC FiO_2 (per 10%)	1.45 (1.02–2.06)	**0.040**	1.23 (0.86–1.75)	0.251
Respiratory rate	0.97 (0.91–1.03)	0.324	0.98 (0.92–1.04)	0.416
Chronic heart failure	1.43 (0.50–4.11)	0.502	1.52 (0.50–4.58)	0.458
Chronic kidney disease	1.29 (0.26–6.38)	0.754	1.07 (0.19–6.12)	0.938

Values are odds ratios (95% CI) from multivariable binary logistic regression for HFNC failure (intubation), including only patients with complete data for all model covariates (APACHE II model:

n = 118, 78 events; SOFA model: n = 106, 65 events). Covariates were identical to the primary imputed models (Table 4). HFNC FiO_2 is expressed per 10% absolute increment. The reference category for awake prone positioning is "no prone positioning." Bold p-values denote $p < 0.05$ (two-sided). Abbreviations: APACHE II, Acute Physiology and Chronic Health Evaluation II; BMI, body mass index; CI, confidence interval; FiO_2, fraction of inspired oxygen; HFNC, high-flow nasal cannula; OR, odds ratio; PaO_2/FiO_2, ratio of arterial oxygen partial pressure to fraction of inspired oxygen; SOFA, Sequential Organ Failure Assessment.

Table S3. Baseline characteristics according to awake prone positioning during HFNC.

Variable	Prone (n = 139)	No prone (n = 83)	*p*	SMD
**Demographics and comorbidities**				
Sex, male	96 (69.1)	52 (62.7)	0.327	0.14
Age, years	61.3 ± 12.0	68.3 ± 15.3	**<0.001**	−0.51
BMI, kg/m◎	25.5 (24.5–27.7)	26.2 (24.7–29.4)	0.221	−0.21
Charlson Comorbidity Index	3.0 (1.0–4.0)	5.0 (3.0–7.0)	**<0.001**	−0.67
Chronic heart failure	44 (31.7)	36 (43.4)	0.078	−0.24
Hypertension	73 (52.5)	43 (51.8)	0.918	0.01
Diabetes mellitus	45 (32.4)	35 (42.2)	0.141	−0.20
Chronic pulmonary disease	28 (20.1)	20 (24.1)	0.489	−0.10
Chronic kidney disease	14 (10.1)	18 (21.7)	**0.017**	−0.32
Cerebrovascular disease	6 (4.3)	9 (10.8)	0.061	−0.25
Malignancy	14 (10.1)	10 (12.0)	0.646	−0.07
Transplant recipient	5 (3.6)	4 (4.8)	0.731	−0.06
**Calendar time**				
Admission order	115 (71–177)	96 (33–161)	**0.048**	0.26
Early era (first 50 admissions)	19 (13.7)	31 (37.3)	**<0.001**	−0.56
**Timing and treatment before ICU admission**				
Symptom onset to ICU admission, days	9.0 (6.0–12.0)	8.0 (6.0–15.0)	0.509	−0.24
Hospital to ICU admission, days	3.0 (0.0–6.0)	3.0 (1.0–8.0)	0.133	−0.32
Corticosteroids	100 (71.9)	40 (48.2)	**<0.001**	0.50
Tocilizumab	21 (15.1)	4 (4.8)	**0.019**	0.35
Favipiravir	118 (84.9)	58 (69.9)	**0.008**	0.36
Hydroxychloroquine	18 (12.9)	28 (33.7)	**<0.001**	−0.51
Azithromycin	19 (13.7)	25 (30.1)	**0.003**	−0.41
Remdesivir	4 (2.9)	0 (0.0)	0.300	0.24
Anakinra	38 (27.3)	17 (20.5)	0.252	0.16
**Illness severity**				
APACHE II	12.0 (9.0–16.0)	17.0 (13.0–21.0)	**<0.001**	−0.65
SOFA at ICU entry	4.0 (3.0–5.0)	5.0 (3.0–8.0)	**0.002**	−0.62
**Hemodynamic and respiratory status**				
Heart rate, beats/min	90.0 (80.0–105.0)	98.0 (82.5–113.5)	0.051	−0.27
Systolic blood pressure, mmHg	131.0 (120.0–150.0)	125.0 (101.2–148.0)	**0.037**	0.36
Mean arterial pressure, mmHg	95.0 (86.7–100.0)	89.2 (75.0–100.0)	**0.017**	0.38
Lactate, mmol/L	1.8 (1.3–2.3)	1.5 (1.2–2.1)	0.146	0.15
Respiratory rate, breaths/min	30.0 (24.0–35.0)	30.0 (19.0–35.0)	0.576	0.13
PaO_2/FiO_2 ratio	89.6 (70.9–112.8)	101.7 (74.2–149.5)	**0.017**	−0.39
HFNC FiO_2, %	80.0 (60.0–95.0)	70.0 (50.0–90.0)	**0.045**	0.32
HFNC flow, L/min	50.0 (45.0–55.0)	50.0 (50.0–60.0)	**0.003**	−0.22
**Laboratory parameters**				
CRP, mg/L	106.0 (43.6–177.0)	136.7 (80.0–201.6)	**0.027**	−0.27
Procalcitonin, ng/mL	0.19 (0.10–0.35)	0.30 (0.13–0.86)	**0.006**	−0.01
D-dimer, ng/mL	1610.0 (840.0–3835.0)	2500.0 (1462.5–4957.5)	**0.003**	−0.11
Ferritin, ng/mL	1064.0 (554.0–1682.2)	824.8 (349.9–1565.0)	0.122	−0.18

Variable	Prone (n = 139)	No prone (n = 83)	*p*	SMD
LDH, U/L	480.0 (388.0–624.8)	411.0 (294.0–588.0)	**0.006**	0.35
Lymphocytes, $\times 10^3/\mu L$	0.50 (0.30–0.70)	0.55 (0.40–0.90)	0.132	−0.14
Pro-BNP, pg/mL	678.1 (273.0–1574.1)	2280.2 (524.0–8909.1)	**<0.001**	−0.47
hs-Troponin T, ng/L	12.1 (6.4–25.7)	34.0 (15.9–95.5)	**<0.001**	−0.40

Data are n (%) for categorical variables and mean ± SD or median (IQR) for continuous variables. Categorical variables were compared with the χ◎or Fisher exact test and continuous variables with the Student t-test or Mann–Whitney U test, as appropriate. Bold p-values denote $p < 0.05$ (two-sided). Admission order denotes the consecutive order of ICU admission; the early era comprises the first 50 admissions. SMD, standardized mean difference (prone minus no prone); absolute values > 0.1 indicate meaningful imbalance. Data were missing for BMI (n = 75), SOFA (n = 79), heart rate (n = 47), systolic blood pressure (n = 43), mean arterial pressure (n = 44), lactate (n = 72), respiratory rate (n = 74), and HFNC FiO_2 (n = 7). Abbreviations: APACHE II, Acute Physiology and Chronic Health Evaluation II; BMI, body mass index; CRP, C-reactive protein; FiO_2, fraction of inspired oxygen; HFNC, high-flow nasal cannula; hs-Troponin T, high-sensitivity troponin T; ICU, intensive care unit; IQR, interquartile range; LDH, lactate dehydrogenase; PaO_2/FiO_2, ratio of arterial oxygen partial pressure to fraction of inspired oxygen; Pro-BNP, pro-B-type natriuretic peptide; SD, standard deviation; SMD, standardized mean difference; SOFA, Sequential Organ Failure Assessment.

Table S4. Sensitivity analyses restricted to patients in whom HFNC was initiated at ICU admission (n = 215; 143 events).

Analysis	Estimate (95% CI)	*p*
**Multivariable logistic regression, APACHE II model**		
Awake prone positioning (yes vs. no)	OR 0.25 (0.11–0.56)	**<0.001**
APACHE II (per point)	OR 1.09 (1.01–1.17)	**0.018**
PaO_2/FiO_2 ratio (per unit)	OR 0.9946 (0.9897–0.9994)	**0.029**
HFNC FiO_2 (per 10%)	OR 1.41 (1.15–1.73)	**<0.001**
**Multivariable logistic regression, SOFA model**		
Awake prone positioning (yes vs. no)	OR 0.26 (0.11–0.60)	**0.002**
Stabilized IPTW	OR 0.24 (0.11–0.52)	**<0.001**
Day-1 landmark, unadjusted (n = 161; 89 events)	HR 0.50 (0.32–0.77)	**0.002**
Day-1 landmark, adjusted (n = 158; 89 events)	HR 0.42 (0.26–0.69)	**<0.001**
Day-2 landmark, unadjusted (n = 132; 64 events)	HR 0.61 (0.36–1.05)	0.073
Day-2 landmark, adjusted (n = 129; 64 events)	HR 0.47 (0.25–0.88)	**0.019**

Models, covariates, imputation (m = 25), and weighting were identical to the primary analyses (Tables 4 and 5). Seven patients in whom HFNC was started after ICU admission (4 after extubation, 3 admitted for non-respiratory reasons) were excluded. Bold p-values denote $p < 0.05$ (two-sided). Abbreviations: APACHE II, Acute Physiology and Chronic Health Evaluation II; CI, confidence interval; FiO_2, fraction of inspired oxygen; HFNC, high-flow nasal cannula; HR, hazard ratio; ICU, intensive care unit; IPTW, inverse probability of treatment weighting; OR, odds ratio; PaO_2/FiO_2, ratio of arterial oxygen partial pressure to fraction of inspired oxygen; SOFA, Sequential Organ Failure Assessment.

Table S5. Sensitivity analyses accounting for calendar time and COVID-19-specific treatments.

Analysis	n (events)	Estimate (95% CI)	p
*Whole cohort, additionally adjusted for calendar time**			
Multivariable model	222 (150)	OR 0.18 (0.07–0.45)	**<0.001**
Multivariable model + corticosteroids, tocilizumab, anakinra	222 (150)	OR 0.17 (0.07–0.44)	**<0.001**
IPTW	222 (150)	OR 0.21 (0.09–0.46)	**<0.001**
Day-1 landmark, adjusted	158 (89)	HR 0.44 (0.26–0.74)	**0.002**
Day-2 landmark, adjusted	129 (64)	HR 0.47 (0.25–0.90)	**0.022**

Analysis	n (events)	Estimate (95% CI)	p
Later era only†			
Multivariable model	172 (119)	OR 0.24 (0.08–0.73)	**0.011**
IPTW	172 (119)	OR 0.22 (0.08–0.59)	**0.003**
Day-1 landmark, unadjusted	123 (70)	HR 0.54 (0.33–0.91)	**0.020**
Day-1 landmark, adjusted	118 (68)	HR 0.48 (0.27–0.87)	**0.016**
Day-2 landmark, unadjusted	109 (56)	HR 0.66 (0.36–1.21)	0.182
Day-2 landmark, adjusted	105 (55)	HR 0.48 (0.23–0.97)	**0.041**
Alternative era boundaries (42nd–60th admission)‡			
Multivariable model	162–180	OR 0.23–0.33	0.008–0.049
Day-1 landmark, adjusted	—	HR 0.48–0.53	0.016–0.040

Estimates refer to awake prone positioning (yes vs. no). Models, covariates, imputation (m = 25), and weighting were otherwise identical to the primary analyses (Tables 4 and 5). *Calendar time was modeled as consecutive admission order using a restricted cubic spline with four knots. †Admissions after the 50th, after corticosteroid use and routine awake proning were adopted. ‡The later era was redefined as starting after the 42nd, 45th, 48th, 50th, 53rd, 56th, or 60th admission; ranges across these definitions are shown. Bold p-values denote $p < 0.05$ (two-sided). Abbreviations: CI, confidence interval; HR, hazard ratio; IPTW, inverse probability of treatment weighting; OR, odds ratio.
